# Cracking the enigma: understanding strigolactone signalling in the rhizosphere

**DOI:** 10.1093/jxb/erad335

**Published:** 2023-08-25

**Authors:** Jed Clark, Tom Bennett

**Affiliations:** School of Biology, Faculty of Biological Sciences, University of Leeds, Leeds LS2 9JT, UK; School of Biology, Faculty of Biological Sciences, University of Leeds, Leeds LS2 9JT, UK; The University of Western Australia, Australia

**Keywords:** Plant–plant interactions, rhizosphere, root exudates, root parasite, strigolactones, symbiosis

## Abstract

The rhizosphere is a complex physical and chemical interface between plants and their underground environment, both biotic and abiotic. Plants exude a large number of chemicals into the rhizosphere in order to manipulate these biotic and abiotic components. Among such chemicals are strigolactones, ancient signalling molecules that in flowering plants act as both internal hormones and external rhizosphere signals. Plants exude strigolactones to communicate with their preferred symbiotic partners and neighbouring plants, but at least some classes of parasitic organisms are able to ‘crack’ these private messages and eavesdrop on the signals. In this review, we examine the intentional consequences of strigolactone exudation, and also the unintentional consequences caused by eavesdroppers. We examine the molecular mechanisms by which strigolactones act within the rhizosphere, and attempt to understand the enigma of the strigolactone molecular diversity synthesized and exuded into the rhizosphere by plants. We conclude by looking at the prospects of using improved understanding of strigolactones in agricultural contexts.

## Teaser

In this review, we examine the multi-faceted roles of strigolactones as signals in the rhizosphere, and try to understand why plants exude so many different types of strigolactone molecules.

## Introduction

### Signalling in the rhizosphere

The rhizosphere is the portion of soil immediately surrounding plant roots. The rhizosphere’s biological richness and chemical complexity, derived from its proximity to plant roots, sets it apart from bulk soil (Berendsen *et al*., 2012). Historically the importance of below-ground biotic interactions in plant growth and survival has been overlooked due to the practical difficulties of underground investigations, particularly in the context of the rhizosphere. More recently, a wealth of studies have bridged this knowledge gap, bringing greater focus into understanding this complex environment. It is vital to agriculture and other related sectors to understand the role that this environment plays in plant physiology.

The rhizosphere acts as a ‘soup’ into which chemicals from plant roots and other organisms can be exuded. These chemicals can then be transported and detected by organisms within the rhizosphere. Many organisms are specialized to live within this environment, forming symbiotic relationships with plants. Termed the ‘rhizosphere effect’, the microbial population density is much greater in the rhizosphere than in surrounding bulk soil (Berendsen *et al*., 2012). Organisms found within the rhizosphere that are of particular interest due to their ability to form symbiotic relationships with a host plant include arbuscular mycorrhizal fungi (AMF), nitrogen-fixing rhizobia, plant-associated bacteria, parasitic plants, pathogenic fungi, pathogenic bacteria, and plant parasitic nematodes (Akiyama *et al*., 2005; Spence and Bais, 2015; Masson-Boivin and Sachs, 2018; Zagorchev *et al*., 2021). In each case, the organism responds to signals in the rhizosphere in order to form a symbiosis with plants. In many cases, this symbiosis is species specific, with symbionts requiring a particular host species. These symbiotic relationships can be mutualistic (beneficial for both the host plant and the symbiont), commensal (where the relationship benefits the symbiont and is neutral for the plant), or parasitic, most probably proving detrimental to the host. From a host point of view, it is not always clear why symbioses are formed, given that certain symbiosis seem to have only detrimental impacts on the host. Despite this, the host maintains its production of exudates that these symbionts rely on to form symbioses. Therefore, the exudates released by the host must play a major role in plant survival, which has a greater positive impact for the plant than the negative impact of organisms eavesdropping on these signals.

This problem is particularly exemplified by strigolactones (SLs), apocarotenoid molecules exuded by plants that act as prolific rhizosphere signals, promoting both positive and negative symbioses. In this review we will discuss the specific role played by SLs in rhizosphere signalling. We will first discuss the nature and formation of symbiotic interactions driven by SLs, and whether the symbionts are the intended targets of these signals, aiding in host plant survival; or eavesdroppers, taking advantage of signals emitted for other reasons. We will then discuss what we know about the signalling mechanisms for these symbiotic interactions, and whether there is specificity in SL signalling within the rhizosphere.

## Strigolactones as intentional rhizosphere signals

**Fig. 1.**
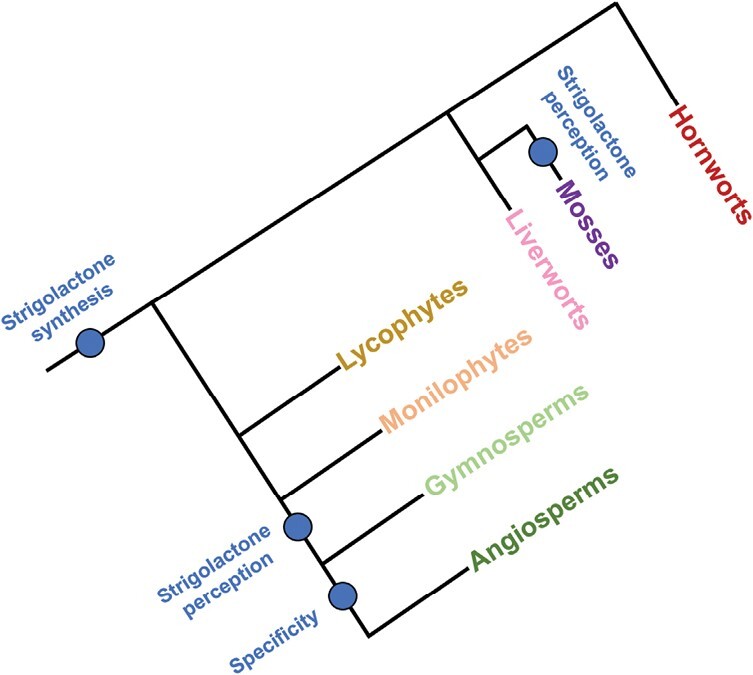
Evolution of strigolactones in land plants. Simplified land plant phylogeny showing the emergence of strigolactone signalling, perception, and specificity in major land plant groups. Strigolactone synthesis is common to all land plant groups, but internal strigolactone perception seems to be unique to mosses and seed plants (gymnosperms and angiosperms). Fully distinct strigolactone signalling is only seen in flowering plants.

SLs were first identified as soil chemicals involved in stimulating the germination of the parasitic plant *Striga lutea* (Cook *et al*., 1966). Subsequently they have been identified as playing a vital role in the recruitment of AMF into symbiosis with plant roots (Akiyama *et al*., 2010) and further as endogenous plant development regulators (hormones) (Gomez-Roldan *et al.*, 2008; Umehara *et al*., 2008). Analysis shows that all major land plant taxa possess the core enzymes required for SL synthesis and, where assessed, all have the capability to produce SL molecules (Walker *et al*., 2019; reviewed in Wheeldon and Bennett, 2021) (Fig. 1). However, the DWARF14 (D14) receptor family is only present in seed plants, and the SMAX1-LIKE7 proteolytic targets which are the specific signalling targets for SLs are only present in flowering plants (Walker *et al*., 2019) (Fig. 1). Hornworts and liverworts are unlikely to have SL receptors (Bythell-Douglas *et al*., 2017; Walker *et al*., 2019), although mosses may have convergently evolved receptors (Lopez-Obando *et al*., 2021); the status of SL signalling in lycophytes and ferns is effectively untested (Fig. 1).

**Fig. 2.**
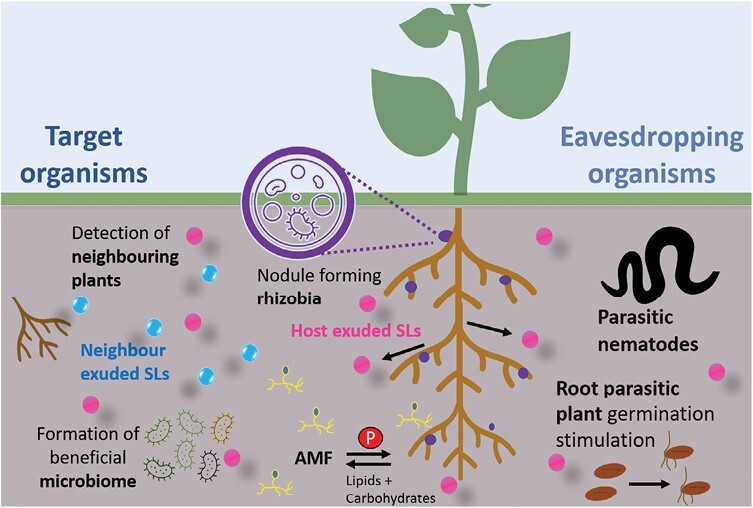
Strigolactone-mediated signalling in the rhizosphere. Summary of strigolactone-mediated interactions with both target and eavesdropping organisms in the rhizosphere.

Given this information, it is therefore very likely that the ancestral role of SLs in land plants was as exuded signals, and that they were later recruited as hormonal signals in seed plants (Walker *et al*., 2019), consistent with the need for new pathways for long-distance signalling in vascular plants (Wheeldon and Bennett, 2021). Given that plant–AMF interactions have been shown to be ancient within the land plant lineage (Remy *et al*., 1994), and are found in all land plant taxa apart from mosses, it is highly likely that the original function of SLs was as exuded signals to promote AMF symbioses. Analysis of SL synthesis and signalling in the liverwort *Marchantia palacea* strongly supports this model (Kodama *et al*., 2022). The question is: are exuded SLs only signals to AMF? In this section, we review the apparently intentional uses of SLs as rhizosphere signalling molecules (Fig. 2).

### Strigolactones and arbuscular mycorrhizal fungi

AMF are a group of monophyletic soil-borne fungi in the phylum Glomeromycota, that are obligately biotrophic, relying on a host plant to complete their life cycle. AMF form symbioses with >80% of extant land plants (Smith and Read, 1997). In this symbiotic relationship, AMF provide the plant with minerals, in particular phosphate, and in return AMF are supplied with lipids and carbohydrates, allowing the host plant to prosper in unfavourable environmental conditions. AMF spores may germinate in the absence of a host plant, but only limited hyphal growth will occur and, if a host plant is not found, growth stops after 25–30 d (Harrier, 2001). However, when a host is present, AMF hyphae have extensive branching near to the host roots (Giovannetti *et al*., 1993). This extensive branching indicates that hyphal growth is important for symbiosis, creating contact with the host root and ultimately allowing for root penetration by the AMF. It was found that SLs are host plant-derived signals that promote induced extensive hyphal branching in germinating AMF spores of *Gigaspora margarita* (Akiyama *et al*., 2005), and which therefore act as a key molecule in the formation of AMF symbioses.

In conditions of low mineral nutrition in the soil, the expression of SL synthesis and transport genes is strongly up-regulated, and the quantity of SLs produced in the roots is dramatically increased (Yoneyama *et al.*, 2012; Kodama *et al*., 2022), as is their exudation to the rhizosphere (Kretzschmar *et al*., 2012). While this up-regulation of SL was initially observed principally in response to phosphate starvation (Yoneyama *et al.*, 2012), it is now clear that SL synthesis is also strongly promoted in the roots in response to nitrate starvation (Sigalas *et al*., 2023). This increased exudation promotes the formation of AMF symbioses, which in turn help to alleviate the phosphate and nitrate deficiency encountered by the plant. In this way, the plant ‘chooses’ when to invest resources in the formation of AM symbioses through very deliberate signalling to potential partners.

### Rhizobia–legume symbioses

In addition to AMF, SLs have also been implicated in the formation of mutualistic rhizobia–legume symbioses. Rhizobia are a paraphyletic group of nitrogen-fixing bacteria (which include many but not all members of the genus *Rhizobium*) capable of reducing dinitrogen into ammonia when in symbiosis, but which cannot independently fix nitrogen. In order for this symbiosis to occur, rhizobia invade the roots of the host legume plants, which in response produce ‘nodules’, organs specialized for nitrogen fixation. Rhizobia are protected within the nodule and are supplied with carbohydrates from the plant, and in return provide the plant with ammonia (Peláez-Vico *et al*., 2016).

Soto *et al*. (2010) first described the possible role of SLs in rhizobia–legume symbiosis, finding that the SL analogue GR24 resulted in increased nodulation in alfalfa (*Medicago sativa*) when inoculated with *Sinorhizobium meliloti*. They also found that this increase in nodulation was not linked to the stimulatory effect of GR24 on *S. meliloti* as there was no increase in *nod* gene expression (Soto *et al*., 2010). Subsequently, it has been established that SLs are not essential for nodule development but may regulate nodule number (Foo and Davies, 2011; Liu *et al*., 2013). This is supported by analysis of SL-deficient *rms1* pea mutants which implicated SLs as positive nodulation regulators required for optimal nodule number formation, but not for nodule formation *per se* (Foo and Davies, 2011). However, the precise role of SLs in rhizobia–legume symbiosis is still unknown. Tambalo *et al*. (2014) found that rhizobia swarming behaviour was influenced by signals from host plants, and identified SL as a possible signalling molecule for this effect in *Rhizobium leguminosarum*. Swarming motility can contribute to rhizosphere colonization (Sánchez-Contreras *et al.*, 2002), and enhanced swarming motility may promote interaction and colonization of plant roots, leading to symbiosis. Tambalo *et al*. (2014) used crude moss extracts to promote swarming, and clear differences were seen in the ability of extracts from wild-type (WT) and SL-deficient plants in the ability to promote swarming, with SL-deficient extracts promoting greatly reduced motility compared with the WT.

### Microbiome assembly

Recruitment of beneficial microbes into the root can improve plant fitness and aid survival in times of nutrient stress. Exudation of SLs has been suggested to regulate or at least modulate plant microbiome assembly because both fungal and bacterial populations have been shown to differ between the WT and SL synthesis mutants. However, the impact of SL on microbiome assembly differs dependent on host species. For example, SL-producing Arabidopsis had a greater fungal diversity than the SL-non-producing *max4* mutant, whereas bacterial communities did not significantly differ (Carvalhais *et al*., 2019). No impact of SL was seen on alpha diversity (richness and evenness) for either fungi or bacteria (Carvalhais *et al*., 2019). In rice, the opposite was seen, with significantly different bacterial communities seen between WT and SL mutants, whereas fungal communities were not significantly different (Nasir *et al*, 2019). In soybean (*Glycine max*), overexpression of SL biosynthesis genes (MAX1d) and signalling genes (D14 and MAX2a) altered the bacterial composition at the individual taxa and community level, but again the fungal community was not significantly different (Liu *et al*., 2020). Furthermore, bacterial communities differed depending on exactly which genes were overexpressed (Liu *et al*., 2020). Kim *et al*. (2022) found distinctive differences in the microbiome composition of 16 rice genotypes that differ in SL exudation under phosphate starvation. Perhaps more interestingly, they found that different SLs with different structures affected different sets of microbes, with orobanchol impacting the relative abundance of the microbes *Burkholderia*–*Caballeronia*–*Paraburkholderia* and *Acidobacteria*, whereas 4-deoxyorobanchol was linked with the *Dyella* and *Umbelopsis* genera (Kim *et al*., 2022). Overall, these data are currently somewhat difficult to reconcile with each other, and clearly more research will be needed in more species in order to understand the role of SLs in microbiome formation. Since Arabidopsis is not characterized as exuding significant quantities of SLs, the significance of these data is particularly difficult to understand. In a general sense, it may be that SLs generally promote host chemotaxis of bacteria and fungi, but that other species-specific factors determine which organisms are ultimately recruited.

### Plant–plant interactions

Recent evidence shows that plants actively detect and respond to neighbouring plants through a variety of mechanisms. Above ground, these mechanisms have been well described, with mechanisms including the detection of reflected light by neighbours (Roig-Villanova and Martínez-García, 2016), touch (Markovic *et al*., 2016), and volatile organic air-borne signals (Karban *et al*., 2013). Plants can also interact with each other below ground; however, these mechanisms are less visible and often difficult to study. Plants release a large amount of organic molecules into the surrounding soil in the form of root exudates to manipulate the rhizosphere (Bais *et al*., 2003) which could theoretically be detected by neighbouring plants.

As known root exudates that also influence plant growth, SLs are obvious candidates to act in neighbour detection, and recent work in pea and rice suggests that this is indeed the case. Wheeldon *et al.* (2022) found that pea SL mutants fail to respond to the presence of neighbouring plants early in their life cycle, and that this is not caused by a failure of SL mutants to respond to other signals in the rhizosphere, but by the lack of SL signals in the rhizosphere itself. In mixed genotype competition experiments, SL synthesis mutants are effectively invisible, and become out-competed by their neighbours, while SL signalling mutants are effectively blind to their neighbours, and grow too large. Using SL synthesis mutants grown with WTs, Wheeldon *et al.* (2022) and Yoneyama *et al.* (2022) both showed that rice and pea both actively take up SLs exuded by their neighbours, and respond by down-regulating SL synthesis and exudation proportionally to neighbour density. The remarkable effect of this is that exuded SL levels in a shared soil volume are constant, irrespective of the number of plants sharing that volume. Taken together, these results suggest that plants use ‘supra-organismal’ SL levels in the soil to adjust their own growth. The early detection and uptake of SLs from neighbouring plants allow a plant to ‘plan’ its long-term growth by mapping its immediate surroundings. However, a plant must also contribute to the supra-organismal SL pool, otherwise neighbour plants will not down-regulate their own growth, and competition will occur. Plants therefore exude SLs as signals to their neighbours, but also use SLs as cues to detect their neighbours, in a ‘live and let live’ strategy in which soil volumes are shared non-competitively by neighbouring plants.

A role for SLs as plant–plant signals has also been suggested by work in the moss *Physcomitrium patens* (Proust *et al*., 2011). Classical work in *Funarium hygrometrica* showed that moss colonies grown adjacent to each other will tend not to grow into each other, implying that there is a diffusible signal that allows them to detect each other, and reduce growth on the facing sides of the colonies (‘Factor H’; references in Proust *et al*., 2011). *Physcomitrium patens ppccd8* SL synthesis mutant colonies will grow into each other, implying that they cannot detect each other normally, but the growth of *ppccd8* colonies can be slowed by an adjacent WT colony producing SLs normally. This suggests that SLs could be Factor H. However, while WT colonies grown with *ppccd8* colonies are suggested to be marginally larger, they still clearly ‘respect’ their *ppccd8* neighbours (Proust *et al*., 2011). Furthermore, the changes that occur in *ppccd8* mutants do not recapitulate all the defined effects of Factor H (Proust *et al*., 2011). More work is thus required to understand the role of SLs as plant–plant signals in mosses. If SLs are plant–plant signals in mosses, it is likely that this is an example of convergent evolution, since the SL perception itself is likely to be convergently evolved (Walker *et al*., 2019).

## Strigolactones as unintentional rhizosphere cues

SLs are exuded by plants as rhizosphere signals to improve their own fitness, usually in greater amounts in times of stress. However, SLs are still exuded in lower amounts under non-stress conditions, probably due to their role in plant–plant interactions (Wheeldon *et al.*, 2022). These SL exudates could therefore act as a ‘cue’ to parasitic symbionts for the presence of a potential host, and are certainly known to do so in some cases. Here we examine to what extent organisms ‘eavesdrop’ on SL signals (Fig. 2).

### Root parasitic plants

Root parasitic plants are those which attach to the root system of a host plant to obtain water, nutrients, and photosynthetic sugar. While there are at least nine groups of root parasitic flowering plants, by far the most numerous and best understood are the witchweeds and broomrapes of the *Orobanchaceae*, which include both facultative and obligate hemiparasites, and obligate holoparasites (Westwood *et al*., 2010; Nelson, 2021). For obligate parasites, the presence of a host plant is essential to seedling establishment, and seed will lie dormant in the soil for decades, awaiting signals from the host root plants that trigger germination. This wait is essential for the parasite to survive as, without a nearby host to attach to acting as a carbon source, the parasitic plant would not survive more than a few days (López-Ráez *et al.*, 2017). Parasitic plants of the *Orobanchaceae* are a major threat to agriculture in Africa in particular, where a heavy infestation can cause crop losses of up to 70% (Parker, 2009; Spallek *et al*., 2013).

SLs were first identified as host-derived signals required for seed germination in *Striga lutea*, and SL-triggered germination occurs throughout the *Orobanchaceae* (Nelson, 2021). These SLs are of course emitted by the host plant for other functional reasons, and are eavesdropped on by the parasitic plants. The range over which these signals are detected is rather low—for instance, *Striga asiatica* seeds must be within 3–4 mm of a host roots in order to germinate, reflecting the maximum root growth which the seedling is capable of (Scott, 2008).

### Parasitic/pathogenic fungi, bacteria, and others

The example of parasitic plants demonstrates that eavesdropping on SL signals is possible—but does this also occur in other groups of symbionts? Given that AMF can detect and respond to SLs, there would certainly be precedent for this in fungi. However, where examined, the evidence suggests that SLs play a largely protective role against fungal parasites. Addition of the SL analogue GR24 strongly inhibited growth of some phytopathogenic fungi, decreasing colony size, and causing an increase in hyphal branching (Dor *et al*., 2011). Other studies have shown that SL does not influence growth of *Pythium irregulare*, an oomycete affecting several cereal and legume crops, with no effect of GR24 seen on growth or hyphal branching *in vitro* (Blake *et al*., 2016).

Aside from potential direct effects of exuded SLs on parasitic organisms themselves, SLs seem to generally have a protective effect against pathogens *in planta*. Torres-Vera *et al*. (2014) proposed that SLs play a role in plant defence through their interactions and regulation of other hormones, including jasmonic acid (JA). They found that SL-deficient tomato mutants were more susceptible to necrotrophic fungal pathogens than the WT, which was caused by an indirect involvement of SLs in plant defence responses, resulting in reduced phytohormones levels of JA, salicylic acid (SA), and abscisic acid (ABA) (Torres-Vera *et al*., 2014). Similarly, *Arabidopsis thaliana* SL mutants were found to be hypersensitive to *Rhodococcus fascians*, a biotrophic actinomycete that causes leafy gall syndrome in part through secretion of bacterial cytokinins (Stes *et al*., 2015). Treatment with GR24 and carotenoid cleavage deoxygenase inhibitor D2 showed that SLs antagonize the morphogenic activity of *R. fascians*. In the presence of GR24, leafy gall syndrome phenotype was almost completely removed (Stes *et al*., 2015). It is therefore likely that SLs are mostly protective against pathogens, due to their role in regulating other hormonal signals, thereby indirectly acting in plant defence. The precise role of SLs in plant defence also probably depends on the pathosystem and experimental conditions (López-Ráez *et al.*, 2017).

### Parasitic nematodes

Plant parasitic nematodes (PPNs) can be ectoparasites, semi-endoparasites, or endoparasites, each differing in their mode of plant root colonization, each causing varying levels of cellular damage (Marro *et al.*, 2021). The role of SLs in parasitic nematode infestation remains unclear. SL application results in changes in nematode infestation in a number of host plant species; however, the function of this is more difficult to determine (Escudero-Martinez *et al*., 2019; Lahari *et al*., 2019). Studies have shown contrasting findings; some studies show that SLs positively attract PPN to the plant roots, while other studies suggest that SLs may negatively regulate PPN by promoting interactions with other rhizosphere organisms. For instance, Marro *et al.* (2014, 2018) found that tomato plants infected with PPN had greater AM colonization and higher SL production compared with plants without PPN. Plants may increase SL biosynthesis to increase AM colonization to help protect from PPN, or plants may increase SL biosynthesis because of PPNs taking nutrients, requiring AMF for nutrient replenishment (Marro *et al.*, 2021). It has also been suggested that the crosstalk between phytohormones may result in SL negatively regulating PPN by reducing ABA levels in plants (Nahar *et al*., 2012; Kammerhofer *et al*., 2015).

## The molecular basis for strigolactones as rhizosphere signals

### Strigolactone biosynthesis and diversity

**Fig. 3.**
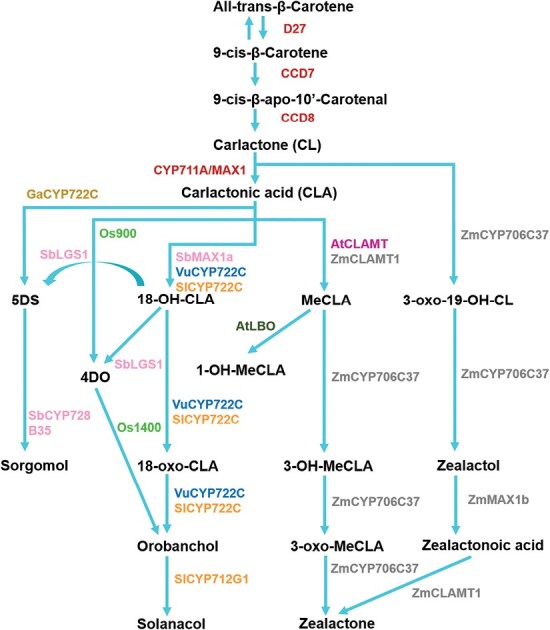
Strigolactone synthesis pathways. Summary of observed strigolactone biosynthesis pathways among flowering plants. Strigolactone molecules and intermediates are in black text, joined by blue lines. Enzymes involved in strigolactone synthesis are coloured according to the species in which they are found. Red, core enzymes in all species; orange, tomato (Sl); gold, cotton (Ga); green, rice (Os); violet, Arabidopsis (At); dark blue, cowpea (Vu); pink, sorghum (Sb); grey, maize (Zm); 4DO, 4-deoxyorobanchol; 5DS, 5-deoxystrigol; MeCLA, methyl carlactonoic acid.

The biosynthesis of SLs begins in the plastid and is initiated when the isomerase enzyme DWARF 27 (D27) catalyses the conversion of all *trans*-β-carotene into 9-*cis*-β-carotene. This is then cleaved by CAROTENOID CLEAVAGE DIOXYGENASE7 (CCD7) into 9-*cis*-β-apo-10ʹ-carotenal and β-ionone (Bruno *et al*., 2014; Felemban *et al*., 2019). CAROTENOID CLEAVAGE DIOXYGENASE8 (CCD8) converts 9-*cis*-β-apo-10ʹ-carotenal into carlactone (CL) and ω-OH-(4-CH3) heptanal (Fig. 3). CL is the common precursor for all SLs, but does not act as an SL itself (Seto *et al.*, 2014). CL is then transported into the cytosol where it is converted into a wide range of different CL derivatives by cytochrome P450 enzymes of the MAX1/CYP711A family, as well as a range of other enzymes (Felemban *et al*., 2019). Understanding the synthesis and function of different CL derivatives remains a key ongoing area of research within the field.

**Fig. 4.**
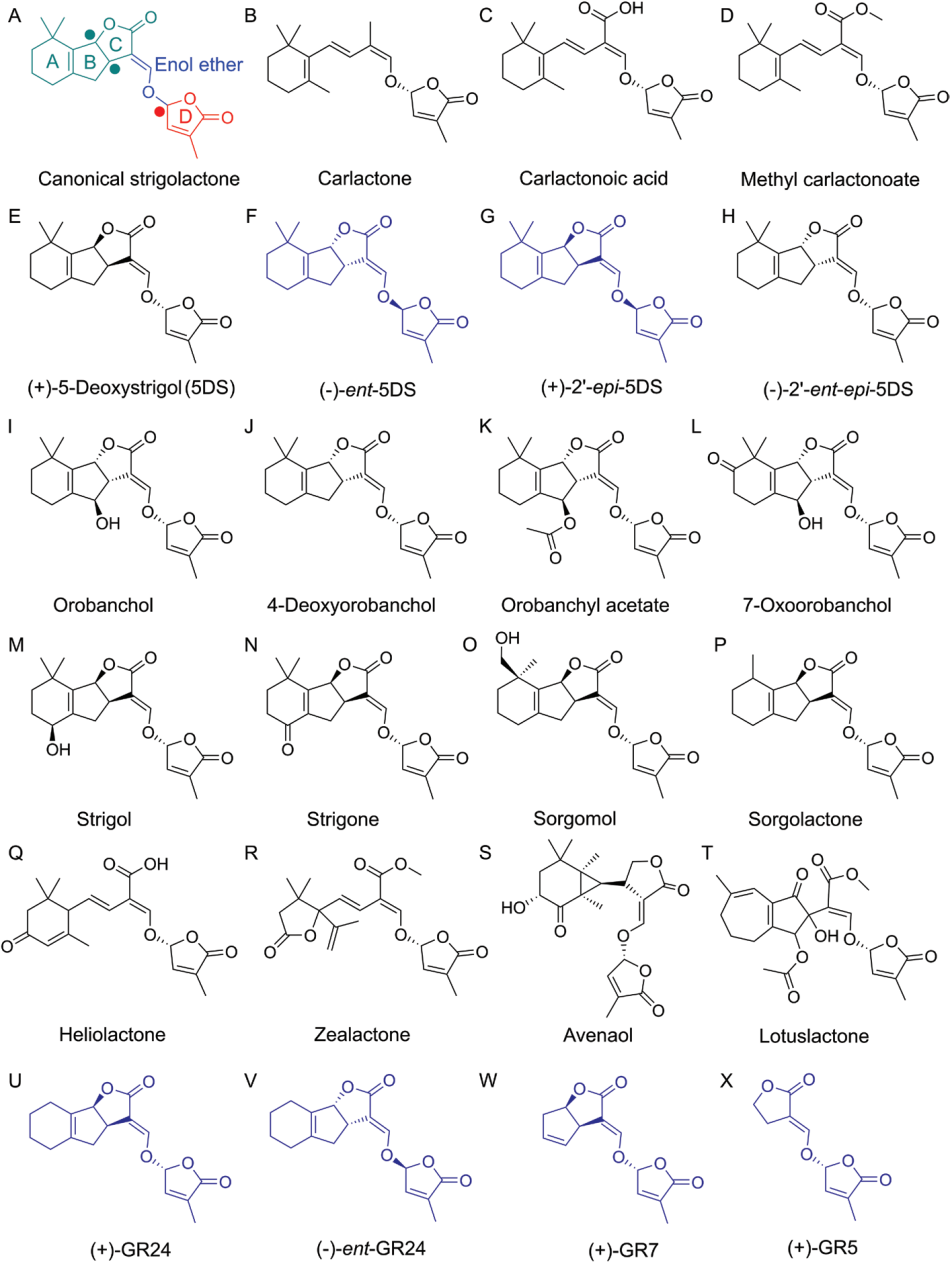
Structures of strigolactones and related molecules. (A) Generic structure of a canonical strigolactone molecule, showing the ABC-rings (green), the enol–ether bridge (blue), and the D-ring (red), with stereocentres marked in red. (B–D) The structures of the strigolactone precursors carlactone (B) and carlactonoic acid (C), along with the non-canonical hormonal strigolactone methyl carlactonoate (D). (E–H) The structures of the four possible stereoisomers of the naturally occurring canonical exuded strigolactone 5-deoxystrigol (5DS) (E). (–)-*ent*-5DS (F) and (+)-2ʹ-*epi*-5DS (G) have the D-ring in the 2ʹS configuration, and are therefore ‘retrolactones’ that do not possess strigolactone activity in plants. (–)-2ʹ-*ent*-*epi*-5DS (H) is the same molecule as 4-deoxyorobanchol (J). (I–L) Members of the orobanchol family of canonical exuded strigolactones: orobanchol (I), 4-deoxyorobanchol (J), orobanchyl acetate (K), 7-oxoorbanchol (L). (M-P) Members of the strigol family of canonical exuded strigolactones: strigol (M); strigone (N), sorgomol (O), sorgolactone (K). (Q–T) Representative non-canonical exuded strigolactones: heliolactone (Q), zealactone (R), avenaol (S), lotuslactone (T). (U–X) Strigolactone analogues with high strigolactone activity in plants but weak activity in AMF: (+)-GR24 (U), (+)-GR7 (W), (+)-GR5 (X), and a synthetic retrolactone, (–)-*ent*-GR24 (V), which has strigolactone-like activity in AMF but not in plants.

The CL derivatives present in each plant species varies, with most plant species capable of producing multiple different SLs (Al-Babili and Bouwmeester, 2015; Yoneyama *et al.*, 2018b). There are at least 32 known SLs thus far discovered, which are classified into two distinct groups, canonical and non-canonical, dependent on their chemical structures (Fig. 4) (Yoneyama *et al.*, 2018b). Canonical SLs consist of a tricyclic lactone ABC-ring system connected via an enol–ether bridge to a methylbutenolide D-ring (Al-Babili and Bouwmeester, 2015), whereas non-canonical SLs lack the characteristic ABC-ring. The enol–ether-linked D-ring is present in both types, and is essential for biological activity in plants (Yoneyama *et al.*, 2009, 2018b; Akiyama *et al*., 2010; Xie *et al*., 2010). There are two stereochemical centres in canonical SLs; at the junction of the B- and C-rings, and in the enol–ether bridge of the D-ring (Fig. 3). Canonical SLs can have either configuration at the B/C stereocentre, which is not important for their activity in plants; non-canonical SLs lack this stereocentre. All natural SLs have the 2ʹR configuration at the D-ring stereocentre, and this is essential for activity in plants. Conversely, the non-naturally occurring 2ʹS configured ‘retrolactone’ version of a given SL will not trigger SL signalling in plants (reviewed in Machin *et al*., 2020).

The model plant *A. thaliana* does not produce canonical SLs in appreciable quantities, only non-canonical SLs, and therefore represents a simple system in which to start understanding post-CL synthesis of SLs (Fig. 4). The first cytosolic synthesis step in Arabidopsis is the conversion of CL to carlactonoic acid (CLA) by MORE AXILLARY BRANCHING1 (MAX1), a CYP711A enzyme (Abe *et al*., 2014). CLA is then converted to methyl-CLA (MeCLA) by a CLA METHYLTRANSFERASE enzyme (CLAMT) (Mashiguchi *et al*., 2022), and then to 1-hydroxymethyl-CLA (1ʹOH-MeCLA) by LATERAL BRANCHING OXIDOREDUCTASE (LBO), a 2-oxoglutrate/iron-dependent dioxygenase enzyme (Brewer *et al*, 2016). Both MeCLA and 1ʹOH-MeCLA are active SLs, but 1ʹOH-MeCLA is the predominant form of SL in Arabidopsis (Abe *et al*., 2014; Yoneyama *et al.*, 2020).

Other species that produce canonical and non-canonical SLs have correspondingly more complex synthesis pathways (Fig. 4). In rice, which synthesizes both canonical and non-canonical SLs, there are four functional MAX1 paralogues which catalyse different reactions from each other. MAX1-Os900 catalyses a double reaction of CL to the canonical SL 4-deoxyorobanchol (4DO) via CLA, while MAX1-Os1400 catalyses the synthesis of orobanchol from 4DO (Yoneyama *et al.*, 2018a). Os900 also probably catalyses the synthesis of non-canonical SLs in rice, including 4-oxo-MeCLA, from putative intermediates including 4-oxo-hydroxy-CL and 4-oxo-CL (Ito *et al*., 2022). However, in maize, a relatively close relative of rice within the grass family, a completely distinct set of SLs are produced and exuded, none of which is a canonical SL (Li *et al*., 2023). The synthesis of these molecules requires a MAX1 paralogue, and CLAMT, but also a novel enzyme, CYP706C37 (Li *et al*., 2023). In sorghum, a particularly close relative of maize, the synthesis pathway is different again. Here, a MAX1 paralogue produces non-canonical 18-hydoxy-CLA, which is then acted upon by a unique sulfotransferase enzyme (LGS1) that produces the canonical SLs 4DO and 5-deoxystrigol (5DS) (Yoda *et al*., 2021). A CYP728B enyzyme then allows conversion of 5DS to sorgomol (Wakabayashi *et al*, 2021). In cowpea and tomato, a CYP722C enzyme can convert CLA to orobanchol without a 4DO intermediate (Wakabayashi *et al*., 2019), while in cotton CYP722C enzymes can convert CLA to 5DS (Wakabayashi *et al*., 2020). In tomato, yet another cytochrome P450, CYP712G1, is required for the conversion of orobanchol to solanacol (Wang *et al*., 2022).

### Beyond canonical and non-canonical strigolactones

The repeated innovation in SL synthesis pathways between even closely related species clearly implies an important functional role for SL diversity. This will examined in more detail below, but it is clear that, at a higher level, some of SL diversity relates to the roles of SLs as hormones versus exudates. Analysis of rice *max1-Os900* mutants that do not make canonical SLs shows that they do not have phenotypes associated with lack of hormonal SL signalling, but cannot form mycorrhizal associations, and trigger germination of parasitic plants much less effectively. These data strongly imply that in rice, canonical SLs act as rhizosphere signals, while non-canonical SLs act as hormonal signals (Ito *et al*., 2022; Mashiguchi *et al*., 2022). This is consistent with the lack of canonical SL synthesis in Arabidopsis, which does not exude appreciable SLs, but still has non-canonical SLs and hormonal SLs signalling (Yoneyama *et al.*, 2020).

However, the limitations of the current canonical/non-canonical nomenclature are clearly illustrated by maize, which only produces non-canonical SLs, which act as exudates and presumably hormones, although it is not clear exactly which molecules perform which tasks (Li *et al*., 2023). Recent results suggest that the distinction between canonical and non-canonical SLs, on the basis of their molecular structure, is not necessarily a helpful way to understand the function of SLs. Instead, it might be better to start categorizing SLs as ‘hormonal SLs’ (MeCLA, 1OH-MeCLA, 4-oxo-MeCLA, etc.) and ‘exuded SLs’, with the latter group including both canonical and non-canonical examples. This is the approach we will follow hereafter.

### Strigolactone signalling: plants and parasitic plants

In seed plants, SLs as hormones are perceived by an α/β-hydrolase fold receptor, D14. The details of strigolactone signalling have been extensively studied and are reviewed elsewhere (e.g. Machin *et al*., 2020); for the purposes of the current article, we only need to consider to what ligands D14 receptors are sensitive. Broadly speaking, D14 seems to have a rather broad ligand specificity, in which the presence of a D-ring enol–ether bound to a molecule approximating the size and shape of the ABC-rings is sufficient to activate the receptor (reviewed in Machin *et al*., 2020). Thus, synthetic SL analogues such as yoshimulactone-green (YLG) can bind to and activate D14, despite only vaguely resembling native SLs. The other main consideration is the stereochemistry of the molecules, with the D-ring needing to be connected to the other moiety in a 2ʹR configuration (Waters *et al*., 2017). The non-naturally occuring 2ʹS enantiomers of native SLs (‘retrolactones’) are not able to efficiently bind to and activate D14, even though their ABC groups are identical to the active, native SLs. As far as has been tested, native SLs seem to activate D14-mediated signalling, including canonical SLs such as 4DO and 5DS that do not appear to act as endogenous hormonal SLs (Scaffidi *et al*., 2014; Flematti *et al*., 2016). Since plants can take up exuded, canonical SLs from the rhizosphere and respond to them in a D14-dependent manner, there is also reasonable evidence that D14 endogenously acts as a receptor for exuded, canonical SLs (Wheeldon *et al.*, 2022). It is also notable that D14 is primarily a single-copy gene in flowering plants, ruling out wide subfunctional specialization among D14 proteins for certain SL molecules (although this might occur within certain clades of organisms where D14 is duplicated) (Bythell-Douglas *et al*., 2017). Thus, the role of non-canonical hormonal SLs as hormones seems to arise primarily from the selectivity of root–shoot transport among SLs, rather than because D14 is particularly specialized to perceive non-canonical, hormonal SLs versus exuded SLs.

D14 is a close relative of another α/β-hydrolase fold receptor, HYPOSENSITIVE TO LIGHT/KARRIKIN INSENSITIVE2 (HTL/KAI2), which acts a receptor for an unknown native ‘KAI2-ligand’ (KL) and functions in a distinct set of developmental processes from D14-mediated signalling, included seed germination (Waters *et al*., 2017). In parasitic plants of the *Orobanchaceae*, the copy number of HTL/KAI2 proteins has increased, often dramatically. For instance, Yoshida *et al*. (2019) found the *Striga asiatica* genome to contain 21 KAI2 paralogues, 17 of which are clearly divergent in structure relative to canonical KAI2, and which are highly expressed at the seed and seedling stage (Yoshida *et al*., 2019). Many of these ‘divergent KAI2’ (KAI2d) proteins in parasite species seems to have re-evolved to perceive exuded SLs, rather than KL, with the net effect that seed germination can be triggered by SLs from potential host plants (Conn *et al*., 2015; Toh *et al*., 2015; Tsuchiya *et al*., 2015). If each KAI2d paralogue has specificity for different SLs, then the rapid evolution of the KAI2d clade may have enabled parasites to recognize numerous hosts exuding different SLs (Yoshida *et al*., 2019).

The best-studied parasite, *Striga hermonthica*, has 11 HTL/KAI2 genes. Xu *et al*. (2018) determined the structural basis of ligand specificity by determining the crystal structures of ShHTL1 (a non-divergent KAI2), ShHTL4, ShHTL7, and ShD14 proteins. While the protein structures were generally highly similar, key differences were seen in the structure of the helical cap. In the divergent KAI2 clade containing ShHTL4, ShHTL5, and ShHTL7, Y150 was changed to F150, resulting in a larger binding pocket, reducing steric hindrance to ligands (Xu *et al*., 2018). This subgroup of the divergent clade has previously been highlighted as being highly sensitive to various natural SLs (Toh *et al*., 2015), therefore the change in residue 150 from Y to F allows *S. hermonthica* to recognize multiple SL types at very low concentrations when secreted from a host (Xu *et al*., 2018). Further analysis of ShHTL7 by Wang *et al*. (2021) identified that ShHTL7 has a particularly high affinity for the F-box protein MAX2 compared with other ShHTLs, responding to picomolar SL concentrations to interact with both MAX2 and the transcriptional regulator SMAX1. Similarly, when expressed in Arabidopsis, ShHTL7 interacted with a very low concentration of SLs *in vitro*, rescuing thermo-inhibited germination in response to synthetic GR24, and highlighting very high sensitivity of ShHTL7 to SLs (Takei *et al*., 2023). Arellano-Saab *et al*. (2021) determined that it only takes a three amino acid substitution to convert Arabidopsis KAI2 into a functional KAI2d-type SL receptor. They found that introducing smaller, more polar amino acids into KAI2 resulted in a protein containing a more flexible pocket and lid, increasing pocket volume and elasticity of the protein, allowing for the accommodation of natural SL confirmations such as (2ʹR)-GR24.

Currently, there is no real evidence that different parasite KAI2d proteins are specialized to detect certain SLs. The presence of a large binding pocket in both D14 and KAI2d proteins allows perception of a broad range of SLs, and it is unclear why expressing multiple highly specialized KAI2d proteins would be a better strategy than expressing a single, broad-spectrum KAI2d. Nevertheless, the increased copy number of KAI2d proteins in parasites does tend to suggest that subfunctional specialization must occur among KAI2d proteins—although this does not necessarily need to be at the level of ligand binding. More work is thus needed to understand the proliferation of KAI2d paralogues in parasites and how this might contribute to host specificity.

### Strigolactone signalling: arbuscular mycorrhizal fungi

AMF sensitivity to SLs is generally measured by levels of hyphal branching in AMF after SL application. Using this system, analysis of a wide range of natural SLs, SL analogues, and SL derivatives has defined some of the key structural features required for SL signalling in the AMF *G. margarita*. As in plants, the presence of a D-ring is absolutely required; however, unlike plants, the bond connecting the D-ring to the lactone moiety does not need to be an enol–ether linkage, suggesting that AMF do not cleave SLs during signalling in the same manner as plants (Akiyama *et al*., 2010). Non-canonical SLs generally have strong hyphal-promoting activity, implying that the B- and C-rings are not essential for signalling in AMF (Mori *et al*., 2016). However, the A-ring is very important, and truncated SL analogues such as the GR7 family that lack an A-ring equivalent have very weak activity against AMF. Similarly, the substitution of the dimethylcyclohexane A-ring seen in most natural SLs with a benzene ring in GR24 significantly reduces hyphal branching activity (Akiyama *et al*., 2010). As in plants, SLs of both configurations at the B/C-ring stereocentre are active in promoting hyphal branching in AMF. Unlike plants, however, retrolactones actively promote hyphal branching, in some cases with the same activity as their parent 2ʹR configured strigolactone (Akiyama *et al*., 2010). Perception of SLs in AMF thus has similar but different features to D14-mediated signalling in plants.

Intriguingly, among the naturally occurring canonical SLs tested, there are considerable differences in hyphal branching-promoting activity. Orobanchol and 5-deoxystrigol (5DS) have very strong activity in AMF, with orobanchyl acetate, fabacyl acetate, and sorgolactone having strong activity and strigol and sorgomol only having moderate activity. Thus, not all canonical SLs are equal in their activity in *G. margarita*. It is possible that other AMF may respond to different exuded SLs with different sensitivity from *G. margarita*, but there are currently few data to address this possibility.

### Connecting diversity and perception: is there specificity in strigolactone signalling and detection in the rhizosphere?

At this point, we return to the question of SL diversity, and can ask—what best explains the myriad types of SLs exuded into the rhizosphere by plants? Why have the later stages of SL synthesis repeatedly re-evolved to incorporate new enzymes, and to generate new exuded SL molecules? Why do different plants produce different ‘blends’ of exuded SLs? What is the selective pressure driving these evolutionary innovations?

The most common explanation for exuded SL diversity is the idea that plants are in an evolutionary arms-race with parasitic plants. There is certainly clear evidence for distinct host specificity among parasitic plants (Fernández-Aparicio *et al.*, 2011) and there is certainly evidence for parasitic plants having different sensitivities to different exuded SLs. For instance, the non-canonical exuded SL avenaol from black oat potently stimulates germination of *Phelipanche ramosa* seeds, but only weakly promotes germination of *S. hermonthica* and *Orobanche minor* (Kim *et al*., 2014). Conversely, Xie *et al*. (2019) found that the non-canonical SL lotuslactone strongly elicited germination of *Phelipanche ramosa* and *O. minor* seeds, but that *S. hermonthica* seeds were 100-fold less sensitive. Nomura *et al*. (2013) tested the activity of different naturally occuring SLs (strigol, 5DS, sorgolactone, sorgomol, and orobanchol) on the germination of seeds of *Striga gesnerioides* and *S. hermonthica*. They found that all SLs were able to induce seed germination in *S. hermonthica*, but only a few SLs were able to induce seed germination in *S. gesnerioides* (Nomura *et al*., 2013).

Thus, it can be argued that evolving new forms of SLs allows plant species to continue forming associations with AMF, while at least temporarily evading their parasites, before the parasite evolves counter-measures, such as new variant KAI2d receptor proteins. This is an appealing idea, which could elegantly explain SL diversity, but the evidence is not entirely supportive. For instance, sorghum produces at least five different exuded SLs (strigol, 5DS, orobanchol, sorgolactone, and sorgomol), of which four are potent stimulants for germination of *Striga* species that parasitize sorghum (Wang and Bouwmeester, 2018). Clearly, the diversification and maintenance of SL types within sorghum cannot be explained by host evasion, since all of them efficiently promote germination of the same parasite. Furthermore, a single mutation in the LGS1 enzyme greatly reduces the germination-stimulating ability of sorghum exudates, without compromising mycorrhization (Yoda *et al*., 2021); sorghum could therefore easily evolve to avoid parasitism. Similarly, in maize, at least six different SLs are produced, at least one of which (zealactone) strongly promotes *Striga* germination, but which can be eliminated by a single mutation (Li *et al*., 2023). The blend of exuded SLs produced in sorghum and maize is therefore difficult to explain if parasite avoidance and mycorrhization are the only evolutionary pressures that matter.

Two further points weigh on this matter. Firstly, most exuded SLs are very similar in structure—for instance, from sorghum, strigol, sorgolactone, and sorgomol only differ from 5DS by the addition of a hydroxyl group in the first two and the loss of a methyl group in the latter. Given the very broad specificity of D14 receptors and, where tested, KAI2d receptors in parasitic plants, it is unrealistic to assume that such minor changes to exuded SLs have evolved to evade perception by parasites. At best, such minor changes might slightly reduce perception by parasites, which might be adaptive, but only under very strong selection pressure. If plants have evolved new exuded SLs to avoid parasitism, it would seem reasonable to assume that the new SL species should be distinctively different from ‘standard’ SLs. The rather unusual structures of avenaol, lotuslactone, and zealactone are perhaps more what we might expect from molecules that have evolved to evade hosts, and the differential perception of avenaol by parasitic plants suggests that this is at least partly effective (Kim *et al*., 2014). Overall, parasite evasion strategies do not seem to satisfactorily explain the existence of blends consisting of very similar exuded SLs. A second key point is whether parasitic plants really represent a sufficiently great burden on most plant species to drive the evolution of counter-measures. While devastating in crop monocultures when there has been a build up of seeds in the soil, parasitic plants are not particularly common in the wild. Also the case of sorghum shows that parasite evasion is easily evolvable—but has not evolved. Overall, there is not currently a convincing case that diversification of SLs is driven only by evolutionary pressure from parasites.

An obvious alternative is therefore that the production and exudation of multiple exuded SLs represents the positive need to signal to multiple different symbiotic partners in the rhizosphere. Since AMF, rhizobia, and other plant-associated bacteria have probably evolved SL perception independently, it could be envisaged that plants exude a blend of SLs that contain molecules optimized for signalling to each partner. There is certainly evidence that hyphal branching of *G. margarita* is more sensitive to some SLs than to others (Akiyama *et al*., 2010), for instance. It could also be argued that different plants have different partners among AMF, rhizobia, and plant-associated bacteria, explaining why different plant species produce different blends from each other. However, this argument falls foul of the similarity argument made above. For most plants, exuded SLs are very similar in structure, and it seems implausible that they are perceived with different sensitivity by different symbionts. For instance, rice exudes a blend of 4DO and orobanchol, which differ by a single hydroxl group; it is highly unlikely that these molecules have evolved to target separate symbionts.

There are two further possibilities worth considering. The first is that exuded SL diversity within species is at least partially accidental, and is caused by the exudation of reaction intermediates. The ABCG transporters that act to exude SLs from roots (Kretzschmar *et al*., 2012) probably have a very broad substrate specificity, and have not evolved to distinguish between ‘final’ SL products and the reaction intermediates. Thus, at any point in time, the blend of SLs exuded from roots probably include a subsample of all SL intermediates currently in the cytosol. The more complex the synthesis scheme, the more possible intermediates there are, and therefore the more types of SL will be exuded. For instance, in maize, three enzymes (MAX1, CLAMT, and CYP706C37) can act on CL in more or less any order, resulting in a range of intermediates (Li *et al*., 2023) that might be exuded, and a more complex blend. The range of similar SLs exuded by sorghum could be explained in the same way. In rice, on the other hand, the more simple synthesis scheme results in only 4DO and orobanchol being exuded. However, in none of these cases is there any reason to assume that the exuded SLs are particularly functionally distinct from each other. If each of the SL molecules exuded by a given species is bioactive in the rhizosphere, then there is little selective pressure to improve the specificity of transporters, or to reduce the complexity of the blend. Are we therefore inferring too much meaning in the ‘blends’ of SLs exuded by given species?

The second, non-exclusive possibility is that the different exuded SLs are not necessarily functionally distinct at the point of perception, but at the time and space of synthesis. That is to say, that different exuded SLs are produced at different times to fulfil different purposes. This idea was raised by Wheeldon *et al.* (2022) because of the different regulatory responses of SL synthesis in pea. Synthesis of orobanchol and orobanchyl acetate (OA) in pea was highly responsive to the presence of neighbouring plants, and rapidly reduced in proportion to the number of neighbouring plants sharing the soil volume (Wheeldon *et al.*, 2022). However, synthesis of fabacyl acetate (FA) showed a much less clear response (Wheeldon *et al.*, 2022). It was therefore proposed that orobanchol and OA might be constitutively exuded as plant–plant signals, and are highly responsive to neighbour detection, while FA is exuded specifically under low phosphate conditions as a signal to AMF fungi, and as such is less responsive to plant density. However, this explanation would probably require differential sensitivity to orobanchol/OA and FA between plants and AMF in order to hold true.

While the two possibilities just examined provide plausible explanations for the diversity of exuded SLs within species, they are not satisfactory explanations for the diversity between different species. We can perhaps understand why close relatives sorghum, maize, and rice each exude multiple SL types, but why do they have such different synthesis schemes from each other in the first place? Ultimately, there is currently no single convincing answer to this question; it is perhaps likely that all four explanations proposed here are simultaneously correct, to different extents in different species. Perhaps major shifts in the later stages of SL synthesis are primarily driven by parasite pressure in small ‘founder’ populations of plants; under these conditions, parasitism might act as a strong enough selective pressure to incorporate novel enzymes into the SL synthesis pathway, and produce more exotic SLs such as avenaol that help avoid parasitism. However, when parasite selective pressure is subsequently reduced, plants return to producing and exuding more standard SL types such as 4DO that inadvertantly trigger parasite germination, because these are much more optimal SLs for promoting AMF associations. However, they continue to produce these molecules by their new reaction schemes.

Whatever the explanations behind it, there is no question that the diversity of exuded SL molecules produced by different plants represents one of the most exciting and challenging questions in the SL field. The non-conservation of the post-CL synthesis pathways between different species is remarkable in itself, with apparent constant incorporation of new enzymes to generate new molecules—or even ‘old’ molecules in new ways. The wealth of recent papers on this topic shows the interest this area holds and the number of exciting discoveries being made. But how can this knowledge now be applied?

## Potential applications of strigolactones as rhizosphere signals

SL exudation by crop plants sits at a nexus of several problems and opportunities in agriculture. There is significant interest in boosting the (often limited) ability of crops to form AM symbioses, in order to improve nutrient and water uptake in agriculture (reviewed in Jacott *et al*., 2017), and also interest in being able to manipulate crop microbiomes. Improving SL exudation, both quantity and type, could represent an obvious avenue to do this. On the other hand, there is also a lot of interest in trying to reduce parasitism of crops by identifying low SL-exuding varieties (Yoneyama *et al.*, 2015; Li *et al*., 2023), or to use SL analogues as suicidal germination stimulants for parasitic plants (Logan and Stewart, 1991). In the latter case, the hope is that application of SL to the soil will stimulate parasitic plant seed germination and, in the absence of a host plant, the parasitic seed will eventually die after a few days, reducing seeds in the seed bank before sowing crops. The ability to choose crops based on their SL exudation type and level could allow potential control of AMF formation and microbiome assembly, or help to control parasitic plants, and characterization of both SL exudation type and level among current crop varieties represents a clear short-term goal (Li *et al*., 2023). Further work is also needed to increase the stability of SL analogues for potential application as suicidal germination stimulants, and also to understand SL analogue specificity for a particular target organism, to prevent undesirable negative reactions on the non-target plants or rhizosphere microbes after SL analogue applications (Koltai, 2014).

## Conclusion

As we hope this review amply demonstrates, the exudation of SLs into the rhizosphere has clear benefits for plants, in terms of both recruiting symbiotic partners and signalling their presence to other plants. However, SL exudation also exposes plants to the risk of eavesdropping and parasitism, which in agricultural contexts can be devastating. The distinctive species-specific synthesis and exudation of SLs represents an ongoing puzzle, but also a fascinating research question, and a clear opportunity to better understand signalling in the rhizosphere. Current evidence does not suggest that strigolactone diversity reflects a requirement for signalling to different symbionts, or that it purely reflects an ability to evade parasitic organisms—even though the proliferation of SLs in plants and that of SL receptors in parasites is certainly suggestive in this case. Ultimately, by understanding how and why plants exude such a diversity of SLs, it should be possible to exert greater control over the symbiotic associations formed by crop plants, in turn helping to improve nutrient use efficiency and prevent yield losses, two of the major challenges in modern day agriculture.
